# *Drosophila melanogaster* as a Model Host for the *Burkholderia cepacia* Complex

**DOI:** 10.1371/journal.pone.0011467

**Published:** 2010-07-12

**Authors:** Josée Castonguay-Vanier, Ludovic Vial, Julien Tremblay, Eric Déziel

**Affiliations:** Institut National de la Recherche Scientifique (INRS)-Institut Armand Frappier, Laval, Canada; CNRS - Université Aix-Marseille, France

## Abstract

**Background:**

Colonization with bacterial species from the *Burkholderia cepacia* complex (Bcc) is associated with fast health decline among individuals with cystic fibrosis. In order to investigate the virulence of the Bcc, several alternative infection models have been developed. To this end, the fruit fly is increasingly used as surrogate host, and its validity to enhance our understanding of host-pathogen relationships has been demonstrated with a variety of microorganisms. Moreover, its relevance as a suitable alternative to mammalian hosts has been confirmed with vertebrate organisms.

**Methodology/Principal Findings:**

The aim of this study was to establish *Drosophila melanogaster* as a surrogate host for species from the Bcc. While the feeding method proved unsuccessful at killing the flies, the pricking technique did generate mortality within the populations. Results obtained with the fruit fly model are comparable with results obtained using mammalian infection models. Furthermore, validity of the *Drosophila* infection model was confirmed with *B. cenocepacia* K56-2 mutants known to be less virulent in murine hosts or in other alternative models. Competitive index (CI) analyses were also performed using the fruit fly as host. Results of CI experiments agree with those obtained with mammalian models.

**Conclusions/Significance:**

We conclude that *Drosophila* is a useful alternative infection model for Bcc and that fly pricking assays and competition indices are two complementary methods for virulence testing. Moreover, CI results indicate that this method is more sensitive than mortality tests.

## Introduction

Members of the *Burkholderia* bacterial genus are well known for the versatility of their ecological niches. They were first isolated from the phytosphere where they were found to be pathogenic to plants [1]. However, it is now known that many *Burkholderia* also have developed beneficial interactions with their plant hosts and have considerable ecological importance: several species of *Burkholderia* have proven to be very efficient biocontrol and bioremediation agents [2], [3]. *Burkholderia* species are among the most antibiotic-resistant bacteria associated with human infections [4]. Some species can in fact survive in antimicrobial agent solutions [5], and inside macrophages [6], [7] and free-living amoebae [8]. Within the genus, the *Burkholderia cepacia* complex (Bcc) has channelled a great part of the interest for all these reasons. Furthermore, species from the Bcc are responsible for chronic granulomatous disease [9] and are posing a considerable threat to immunocompromised individuals such as cystic fibrosis (CF) patients. The seriousness of a Bcc infection is highlighted by the fact that CF patients infected with Bcc strains suffer a faster health decline than when infected with *Pseudomonas aeruginosa* [10]. The Bcc is composed of at least seventeen closely related genomic species or genomovars, all of which having been recovered from CF individuals [11], [12]. Their prevalence is however not equal: *B. multivorans* and *B. cenocepacia* are the most encountered, with the latter also associated with the highest mortality rate within the CF community [11].

Much remains to be done to better understand the mechanisms behind the broad virulence of the Bcc, and development of animal models seems therefore inevitable. The traditional murine model has proven useful in the quest for understanding the virulence mechanisms of the Bcc [13], [14], [15], [16], but the search is on for more cost-effective alternatives and for somewhat less controversial widescreen models with faster generation time.

Over recent years, several alternative infection models have been developed for the Bcc, notably *Galleria mellonella*, *Caenorhabditis elegans* and alfalfa (*Medicago sativa*) [17] [18] [19]. The alfalfa seedlings model was the first proposed alternative to mice. This simple model to assess virulence revealed various patterns of infection between Bcc strains [18]. The larvae of the greater wax moth *G. mellonella* has recently been proposed as an useful model for the testing of different strains of the Bcc [17], partly because it had previously shown good correlation between *P. aeruginosa* infection outcomes in mammals and in lower organisms [20]. However, *M. sativa* and *G. mellonella* are not easily manipulated genetically. Hosts with which both reverse and forward genetics are readily possible represent additional advantages. Thus, the nematode *C. elegans*, easily genetically manipulated, has also been suggested [19], but produced mixed results regarding infection outcomes [17], [19], [21].

The genome sequence of the fruit fly has been unravelled several years ago [22] and mutants are readily available. Although *Drosophila* does not possess an acquired immune system, its innate counterpart is very similar to the mammalian one [23], [24]. The fruit fly is capable of cellular as well as humoral responses when faced with invaders: the phagocytosis is done by its plasmatocytes and its fat body produces an array of antimicrobial peptides. The signalling cascades involved in the production of these molecules represent the milestone of the similarity between the innate immune system of vertebrates and of the rest of the animal kingdom [25]. For these reasons, the fruit fly offers great potential to give insights on host-pathogen interactions. In fact, *Drosophila melanogaster* has already proven to be a great tool in the study of plant or fungal pathogens such as *Erwinia carotovora* [26] and *Cryptococcus neoformans* [27], but also of human opportunistic ones, such as *P. aeruginosa* [28].

In this work, we establish the use of the fruit fly as an effective model of infection for not only discriminating species and strains within the Bcc but also for the study of Bcc virulence factors. We also show that use of competitive index provides supplemental discriminating power for the characterization of virulence factors.

## Results and Discussion

### *Burkholderia cepacia* complex does not kill *D. melanogaster* when fed to the fly

Validating the fruit fly as an effective model in the study of the virulence of the Bcc species seemed promising because *Drosophila* was already used successfully with other pathogenic bacteria. Two different methods have been employed to infect *Drosophila* with bacteria: fly feeding, which involves feeding starved flies with bacteria, and nicking, which implies pricking flies in the thorax with a needle dipped into bacterial suspension. For instance, both feeding and pricking infections performed with *P. aeruginosa* are lethal to the flies [28], [29], [30].

Hence, several species from the Bcc were first tested for their capacity to kill fruit flies following ingestion. Interestingly, *B. multivorans* LMG16660, *B. vietnamiensis* LMG 18835, *B. ambifaria* HSJ1, *B. pyrrocinia* LMG21824, *B. cenocepacia* K56-2, *B. cenocepacia* LMG18830, *B. dolosa* LMG21819 and *B. stabilis* LMG18870 were all incapable of producing mortality during the trials (data not shown). Variables that could potentially have an influence on infection outcomes were then modified: flies were deprived of food and water for 9 h instead of 7 h, the initial temperature of 21°C was raised to 25°C, and bacterial concentration on which flies were left to feed was doubled. Still, the flies were not killed by Bcc bacteria using this method. For several feeding assays, the bacterial concentration inside the flies was recorded to verify that the flies had indeed ingested bacteria. For instance, up to 1.9×10^5^ CFU per fly could be recovered for *B. ambifaria* HSJ1 twenty days after the beginning of the infection, without visible adverse effect on the flies.

Since ingested Bcc species are able to colonize the flies without harming them, it is interesting to speculate that these bacterial species could behave as endo-residents of *Drosophila*. To date, however, no finding of any *Burkholderia* species in laboratories flies [31], [32] or in natural populations [33] supports this hypothesis.

### Fly pricking is effective at generating a mortal infection

Given that feeding the flies with Bcc strains did not produce any mortality, assays with the nicking method were conducted.

**Figure 1:**
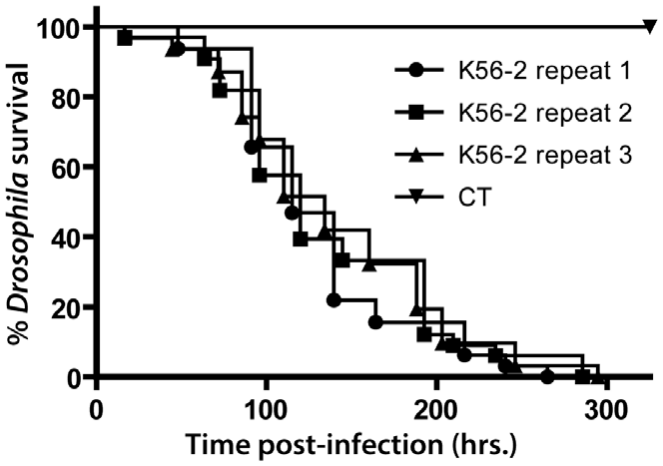
Survival curves for *D. melanogaster* flies challenged with *B. cenocepacia* K56-2. Pricking assays were performed in three independent replicates, each with a minimum of 30 flies. Statistical significance (Log-rank analysis (Mantel-Cox)) between survival curves is shown with *p<0.05 and ***p<0.0005.

Figure 1 shows survival curves for *D. melanogaster* when challenged with *B. cenocepacia* strain K56-2 on three different assays. Results demonstrate that the method can reveal fly killing by a Bcc strain with effectiveness. No statistical difference was observable among survival curves (Log-rank (Mantel-Cox) test), showing that this method is highly reproducible and accurate.

**Figure 2:**
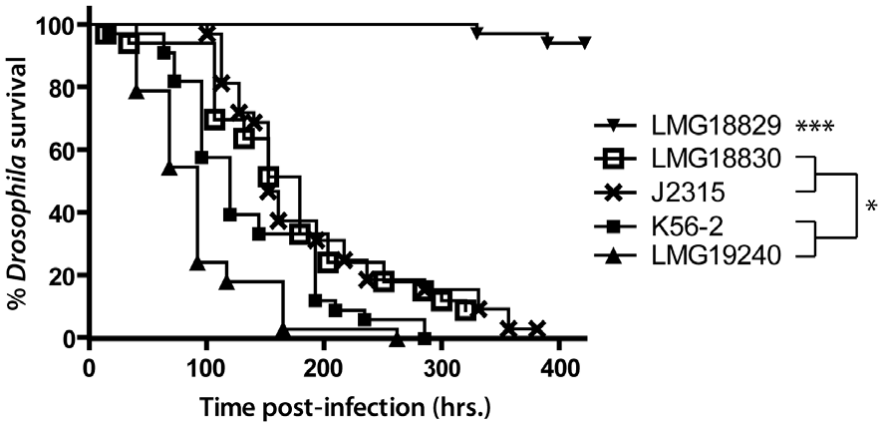
Survival curves for *D. melanogaster* infected with *B. cenocepacia* strains. Pricking assays were performed with a minimum of 30 flies for each strain. Statistical significance (Log-rank analysis (Mantel-Cox)) between survival curves is shown with *p<0.05 and ***p<0.0005.

Pricking experiments uncovered differences in terms of virulence between the Bcc species and strains, allowing discrimination between strains of one particular genomovar but also between Bcc genomovars. As a demonstration, flies were infected with five different wild-type strains of *B. cenocepacia* (genomovar III), revealing variability in infectious capacity between strains of the same Bcc genomovar (Figure 2). Among these strains, we tested K56-2 and J2315 two closely related strains belonging to the epidemic ET-12 lineage [34]. We observed that J2315 is less virulent than K56-2 in the *D. melanogaster* model, which is in agreement with recent data from Uehlinger and colleagues who reported that J2315 is also less virulent than K56-2 in two other alternative models, *G. mellonella* and *C. elegans* [35]. While strain LMG 18830 (CEP511) exhibits moderate virulence in the *Drosophila,* mouse and wax moth models, it produces very different infection outcomes in alfalfa (pathogenic) and *C. elegans* (non lethal) [18], [19].

**Figure 3:**
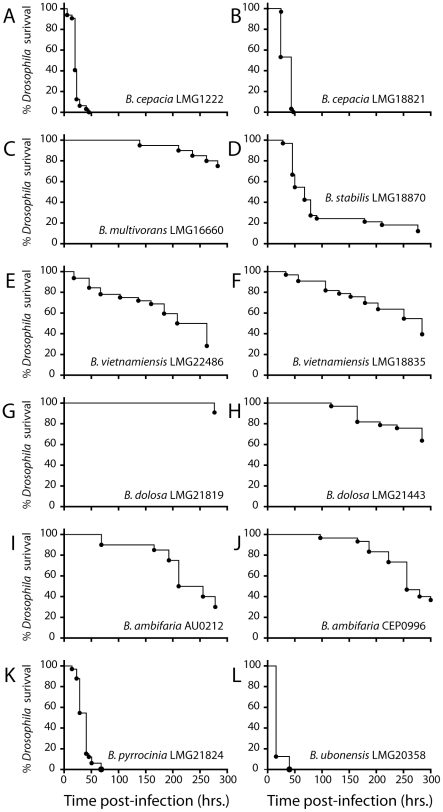
Survival curves for *D. melanogaster* infected with Bcc strains. Pricking assays were performed with a minimum of 30 flies for each strain. **A**: *B. cepacia* LMG1222, **B:** *B. cepacia* LMG18821, **C:** *B. multivorans* LMG16660, **D:** *B. stabilis* LMG18870, **E:** *B. vietnamiensis* LMG22486, **F:** *B. vietnamiensis* LMG18835, **G:** *B. dolosa* LMG21819, **H:** *B. dolosa* LMG21443, **I:** *B. ambifaria* AU0212, **J:** *B. ambifaria* CEP0996, **K:** *B. pyrrocinia* LMG21824, **L:** *B. ubonensis* LMG20358.

A number of other Bcc genomovars were then tested and found to display large differences in virulence toward *D. melanogaster* (Figure 3). *B. cepacia* strains were from different sources (one was first isolated from onions while the other is a clinical strain) and yet, both are among the most virulent strains in the fly pricking model (Figure 3A and B). They kill 100% of the flies within 3 days. These results are similar to what has been obtained with other hosts, murine or alternative models alike. These data obtained with *B. cepacia* illustrate also the environmental isolates appear particularly virulent in alternative infection models, and sometimes more virulent than the clinical isolates as also observed with the *C. elegans* model [19].

*B. pyrrocinia* LMG21824 and *B. ubonensis* LMG20358 kill flies in less than 75 hours, hence being among the most lethal to flies (Figure 3K and L). There are no data in mammalian hosts for these two genomovars; however, they are among the most virulent strains in the *C. elegans* model [19].

*B. stabilis* (Figure 3D), *B. ambifaria* (Figure 3I and 3J) and *B. vietnamiensis* (Figure 3E and 3F) can be classified as intermediate in their virulence towards the fly. These results are consistent with previous work on mammals, on alfalfa and on *G. mellonella* [17], [18]; the only exception being *B. ambifaria* CEP0996, scoring 3 on 3 in pathogenicity tests conducted with *C. elegans* [19].

At the other end of the pathogenicity spectrum, *B. multivorans* LMG16660 (Figure 3C) and *B. dolosa* LMG21819 (Figure 3G) are poorly virulent. The other tested *B. dolosa* strain, LMG21443 (Figure 3H), is slightly more virulent than LMG21819 but still takes more than 12 days to kill only 40% of the flies (Figure 3G and H). The latter *B. dolosa* strain was also avirulent in *C. elegans* [19] while strain LMG21443, more pathogenic to flies, had a LD_50_ of 40,000 CFU in *G. mellonella* [17] and a pathogenicity score of 2 on 3 with *C. elegans* [19].

The results obtained for *B. multivorans* LMG16660 (Figure 3C) are comparable to other works performed with alternative host models. For instance, no or very little mortality was observed with *C. elegans* [19] or alfalfa [18], even with several other strains of that species. *B. multivorans* strains C5393 and C1376 were tested in the rat agar bead model [18] and the animals once again showed very little signs of pathology. In this case, it was assumed to be because of a lower ability of the microorganism to grow in its host. However, poor growth or persistency of the bacteria cannot explain the lack of pathogenicity of *B. multivorans* towards flies. Indeed, 6.55×10^6^ CFU per fly were recovered on day 8 following a septic injury. Experiments performed on *Panagrellus redivivus*, a nematode capable of surviving several days at 37°C revealed that *B. multivorans* strains were only able to kill the model at 37°C but not at 25°C suggesting that this genomovar carries virulence functions upregulated at 37°C [36].

Overall, there is a very good correlation between the results obtained in the fly pricking assay and those obtained with alternative or murine hosts.

### *Drosophila* mortality correlates with bacterial growth and persistence *in vivo*

**Figure 4:**
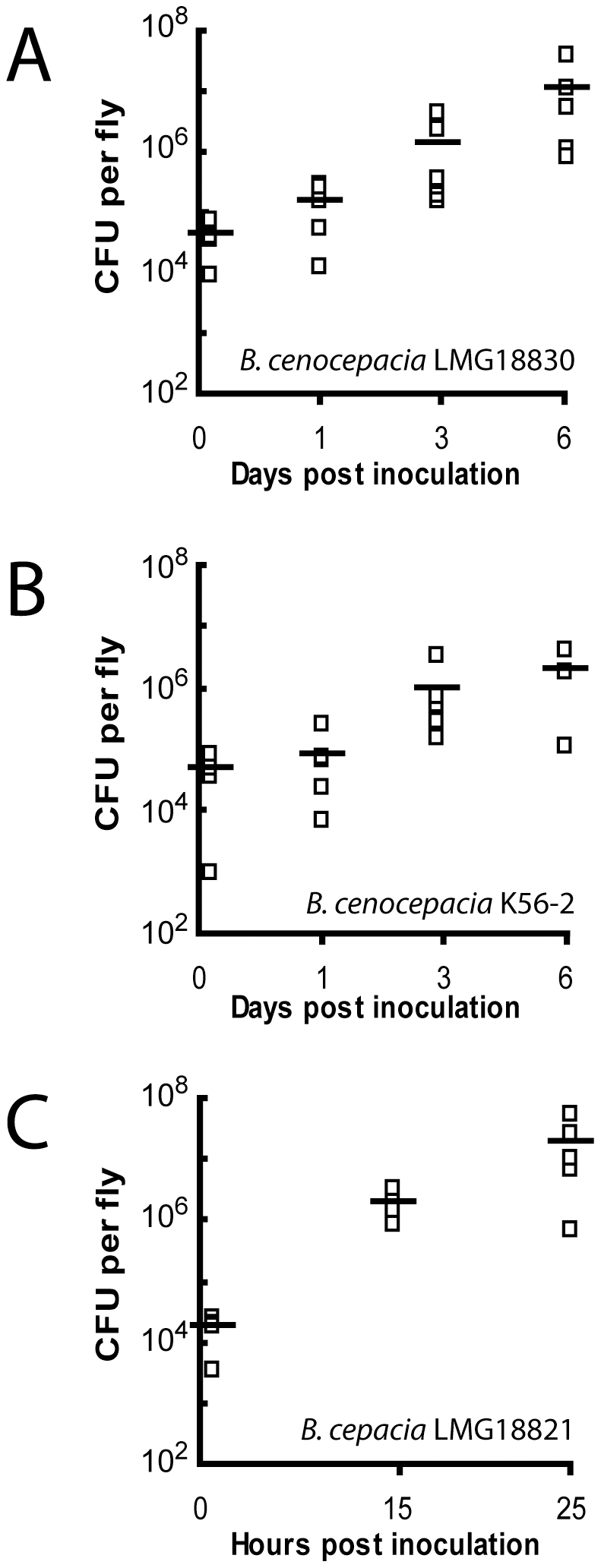
Relative bacterial load kinetic of fruit flies infected with various Bcc species. At the indicated time points, bacterial load was quantified from living fruit flies as described in Materials and Methods. **A**: *B. cenocepacia* LMG18830, **B**: *B. cenocepacia* K56-2, **C**: *B. cepacia* LMG18821.

To verify the presence of bacteria in pricked flies throughout the infections, bacterial survival *in vivo* was measured for three Bcc strains. Figure 4 demonstrates that every strain tested was capable of colonizing the fly and able to replicate inside the host, although with different rates.

The CFU/fly for the three strains measured one hour post infection were all equivalent: 4.55×10^4^±2.73×10^4^ CFU per fly were recovered for *B. cenocepacia* LMG18830 while it was 4.63×10^4^±3.15×10^4^ CFU per fly for *B. cenocepacia* K56-2 and 1.73×10^4^±8.26×10^3^ CFU per fly for *B. cepacia* LMG18821. However, while it only took 1 day for *B. cepacia* LMG18821 CFUs to reach 10^6^ CFU/fly, approximately 6 days were needed for *B. cenocepacia* LMG18830 to reach the same CFU number in the flies. Accordingly, *B. cepacia* LMG18821 kills flies much faster than *B. cenocepacia* LMG18830 or K56-2; all LMG18821-infected flies died in less than 50 hours whereas approximately 90% of the flies for K56-2 and LMG18830 were still alive at that time during infection. These results support the hypothesis that strains displaying slower *in vivo* growth rates also kill flies more gradually.

### Fly mortality when infected with Bcc mutants

An effective alternative infection model should allow discrimination between virulent and avirulent bacterial strains. Since the pricking assay revealed conclusive, mutants of *B. cenocepacia* strain K56-2 previously reported to display reduced virulence towards mammals were thereby examined in the fly (Table S1). The *in vitro* growth rates of the mutants had been determined to be the same than the wild-type (data not shown).

**Figure 5:**
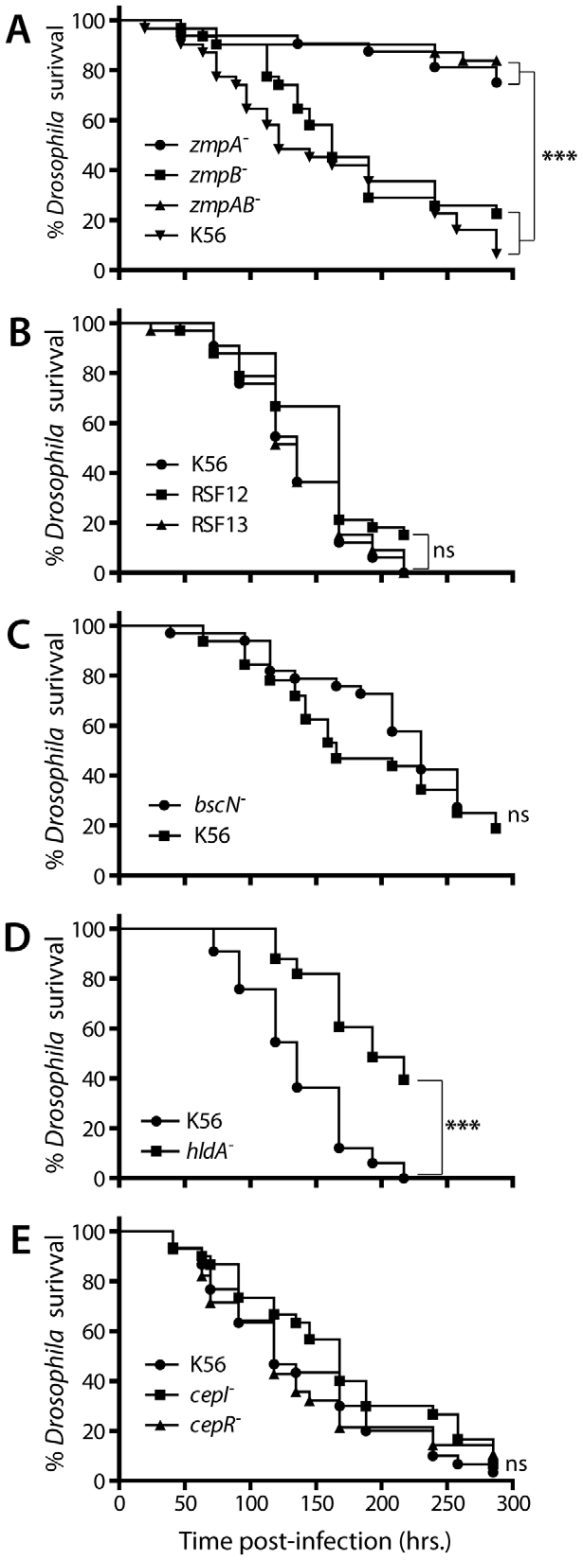
Survival curves for *D. melanogaster* infected with mutants of *B. cenocepacia* K56-2. The killing ability of wild-type *B. cenocepacia* K56-2 was compared to several mutants: **A:** *zmpA^-^*, *zmpB^-^* and *zmpA^-^zmpB^-^*, **B:** RSF12 and RSF13. **C:** *bscN^-^*, **D:** *hldA^-^*, **E:** *cepI^-^* and *cepR^-^*. Pricking assays were performed with a minimum of 30 flies for each strain. Statistical significance (Log-rank analysis (Mantel-Cox)) between survival curves is shown with ***p<0.0005 and ns  =  non-significant.

Two zinc-dependent metalloproteases, ZmpA and ZmpB, known to cleave several proteins important in host defence, are clearly involved in the virulence of *B. cenocepacia* K56-2. Indeed, a *zmpA* mutant is less virulent in a rat chronic respiratory infection model [37], as is also the *zmpB* and the double *zmpA zmpB* mutants [38]. However, in *C. elegans*, *G. mellonella* and alfalfa, *zmpA* and *zmpB* mutants are as virulent as the wild-type K56-2 [35]. Significantly, in our fly pricking model the *zmpA* mutant is significantly less pathogenic than the wild-type strain, while the *zmpB* mutant does not show a reduced pattern of mortality (Figure 5A). Moreover, infections performed with the *zmpA*-*zmpB* double mutant produce survival curves very similar to the ones obtained with the *zmpA* mutant. One possible explanation for the lack of contribution of ZmpB in the fruit fly is that this protease is only active when the temperature reaches 28°C [38]. Indeed, our pricking experiments were conducted under a controlled temperature of 25°C. However, the report that a *zmpB* mutant was not attenuated in the *G. mellonella* and alfalfa models in experiments performed respectively at 30 and 37°C does not support this hypothesis [35].

Another protease HtrA is required for survival to environmental stress [39]. *B. cenocepacia* K56-2 strains RSF13 and RSF12 are deficient for the production of this protease (mutation in BCAL2829 gene encoding for the HtrA protease and mutation in a two-component regulatory system, respectively). Experiments performed in the rat agar bead model showed these two strains cannot survive *in vivo* during lung infections [39]. However, we observed no difference for these two strains compared to the virulence of the wild-type strain in *Drosophila* survival experiments (Figure 5B). A result also obtained with *C. elegans*, *G. mellonella* and alfalfa (Table S1)[35].

Bcc, as a wide range of bacterial pathogens, utilizes a type III secretion system to deliver virulence proteins directly into target host cells. BscN is a Type III secretion system ATP-binding protein that likely generates energy for the secretion of virulence proteins [14]. In the mouse agar bead model, CFU recovered from the lungs and spleens were significantly lower for *bscN* mutants [14]. Our trials conducted with the *bscN* mutant of strain K56-2 shows a partial difference in fly mortality pattern when compared to the parental strain (Figure 5C). This experiment, performed in triplicate, was repeated on two occasions and each time produced similar results: a subtle pathogenicity lag for the *bscN* mutant about mid-time post-infection. Interestingly, a *bscN* mutant of *B. cenocepacia* H111 also gave mixed infection outcomes when performed on *C. elegans*: difference between wild-type and *bscN* mutant could only be observed at particular time-points post-infection [21].

The role of LPS as a virulence factor of *B. cenocepacia* has been tested using strain SAL1 defective in the expression of *hldA* and *hldD* genes, which code for enzymes involved in the synthesis of complete LPS core oligosaccharides [40]. Results in the rat agar bead model of chronic lung infection showed a reduced infectious capability for the SAL1 strain [40]. The rats had in fact completely cleared the mutant two weeks after infection. We also found SAL1 to be attenuated in the *D. melanogaster* infection model (Figure 5D). Decreased virulence of this mutant was also observed in *G. mellonella* and *C. elegans* (Table S1)[35]. Mutations in LPS structure make the cells more sensitive to antimicrobial peptides, a key component of the host innate immune defense response, notably in *Drosophila* [36]. Thus an increased susceptibility to antimicrobial peptides could explain the results obtained with strain SAL1.

The *cepI* and *cepR* genes are part of a quorum sensing system widely found in Bcc species [41]. These genes affect the expression of various virulence factors, notably the zinc-dependent metalloproteases ZmpA and ZmpB [41], [42]. Both *cepI* and *cepR* mutants have shown a decrease in mean percentage of lung inflammation in the rat agar bead infection model when compared to wild-type K56-2 [42]. In our fly pricking model, the *cepR* and *cepI* mutants are not attenuated (Figure 5E). Accordingly, the *cepI* mutant of K56-2 is as virulent as the wild-type strain in two infection models: *G. mellonella* and alfalfa. However, the same mutant showed reduced killing of *C. elegans* [35]. This divergence from the other infection models is probably due to AidA, a protein regulated by quorum sensing and one of the major virulence factors for *C. elegans* pathogenicity [43].

### Competitive index assays

Usually, virulence with alternative models is evaluated in terms of lethality (time, or number of bacteria, LD_50_, required to kill the infected host). Some information may however be lost in the process. Also, if a gene mutation does not increase the mortality rate of the flies, it does not necessarily mean that the gene product does not play a role in the virulence of the bacterium. CI is a well-established, sensitive method to examine bacterial virulence in mammalian host such as the mouse or the rat [44], [45]. These assays provide information on the capacity of a mutant strain to compete *in vivo* with the wild-type strain. For several genes investigated in our pricking assays (e.g. *hldA* or *htrA*), their role as virulence determinants had previously been demonstrated by CI analysis in the rat agar bead model [39], [40].

To provide enhanced sensitivity and discriminating power to the fly mortality tests, CI was adapted to be performed in *Drosophila* (see Materials and methods for details). The CI is defined as the ratio between the mutant strain and the wild-type in the output (96 h post-infection) divided by the ratio of the two strains in the input (inoculum).

**Figure 6:**
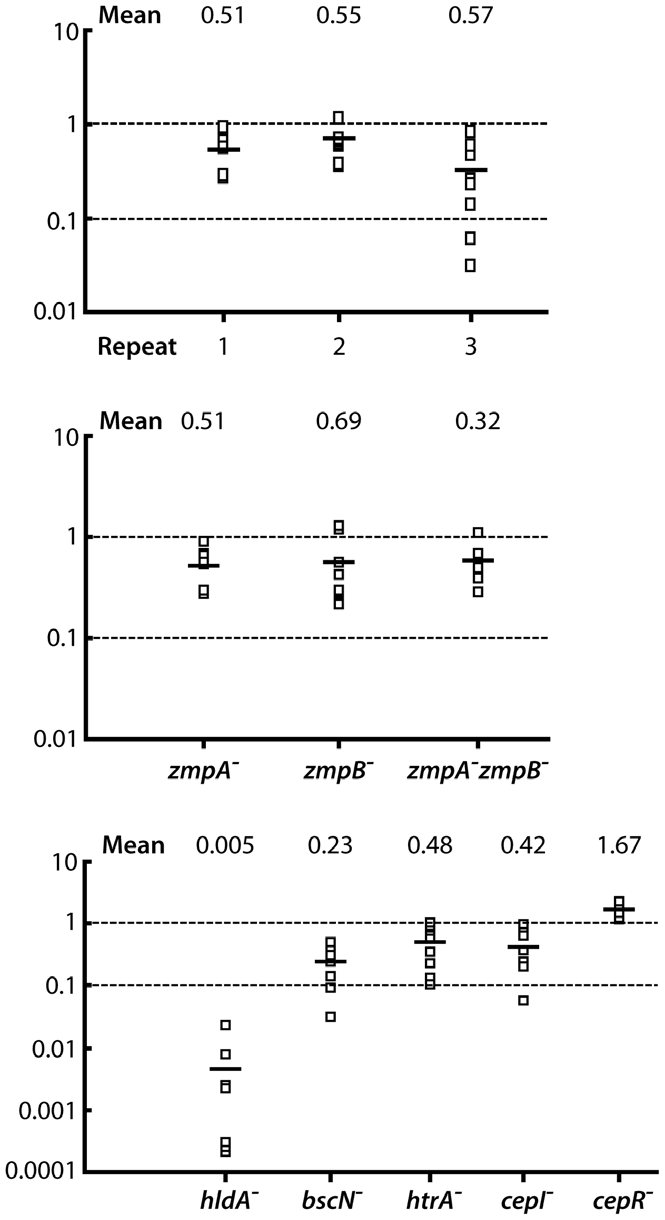
Competitive index (CI) analysis of *B. cenocepacia* mutants in the *D. melanogaster* model. CI is defined as the ratio between the wild-type K56-2 and the mutant in the output (bacteria recovered from the fruit fly 96 h post infection) divided by their ratio in the input (inoculum). Each empty square represents the CI value obtained for one fly. A CI of less than 1 indicates a virulence defect. The mean of the CI is shown as a solid line. **A**. Three independent CI analyses performed with *zmpA* mutant. **B**. CI analyses of *zmpA*, *zmpB* and *zmpA zmpB* mutants. **C**. CI analyses of *hldA*, *bscN*, BCAL2831, *cepI* and *cepR* mutants. For *htrA^-^*, the CI was determined with strain RSF12 containing a mutation in the BCAL2831 gene.

In preliminary studies, we observed that the bacterial concentration used as inoculum or the choice of the time point for the output have no effect on the CI value (data not shown). Figure 6A shows three independent tests performed with the *zmpA* mutant that all produced very similar mean indices, confirming the reproducibility of the approach. For each experiment, the small variability between individual indices also highlights the precision of the results and further validates the method. In every case, mean CIs were all below 1, which indicates that the *zmpA* mutant is less competitive than the wild-type *in vivo*.

During the pricking experiments, the *zmpB* mutant could not be differentiated from its parental strain (Figure 5A). However, the CI results revealed that this mutant is actually less competitive than the wild-type (Figure 6B). It was also hard to differentiate the *Drosophila* survival curves of *zmpA* from those of the double mutant *zmpA zmpB*. CI assays unravelled a difference between the strains: as expected, the double *zmpA zmpB* mutant proved less competitive than the single *zmpA* or *zmpB* mutants.

Similarly, CI assays were performed with the RSF12 strain and revealed that this mutant is less competitive than the wild-type (Figure 6C). This result is in concordance with those previously reported for rats by Flannagan *et al.* [39]. In fact, in this latter study, conclusions regarding virulence were only based on CI where both RSF12 and RSF13 could not compete against the parental strain K56-2 and were completely cleared by the rats [39]. Our data with CI in *D. melanogaster* confirm that HtrA is involved in bacterial survival *in vivo*.

Mortality assays performed with the *bscN* mutant showed only a partial difference with K56-2 wild-type (Figure 5C). In contrast, CI experiments clearly exposed the weakness of the *bscN* mutant (Figure 6C). The CI tests shed more light on the importance of type III secretion system in the pathogenesis of Bcc. CI performed with the mutant *hldA* (strain SAL1) produced the lowest CI observed, with a low mean result of 0.005 and a dramatic defect in *in vivo* survival. This result also highlights data obtained with the rat agar bead model of chronic lung infection [40].

Finally, the *cepI* mutant is less competitive than the wild-type, with a mean value of 0.42 for the CI. Interestingly, we obtained a value of 1.67 for the *cepR* mutant, which indicates that this mutant is more competitive than K56-2 wild-type in the fruit fly (Figure 6C). In *B. cenocepacia*, CepR functions both as a positive and negative regulator of virulence factors [46]. One possibility is that CepR downregulates genes required for *in vivo* survival. CI in mammalian host has, to the best of our knowledge, never been performed with *cepI* or *cepR* mutants. Thus, a *cepR* mutant would be more competitive than the wild-type in mouse or rat models.

Taken together, not only CI experiments confirm results obtained with survival curves (e.g. for *zmpA* and *hldA* mutants) but also reveals new information not detectable by mortality tests (e.g. with *zmpB*, *bscN* or *cepR* mutants). We show for the first time that CI analysis can be used with invertebrate model hosts with results in accordance with those obtained with mammalian models.

### Conclusion: potential of the *Drosophila* model

Several pathogens express virulence functions only above specific temperatures. Unlike *G. mellonella* or the murine model, both *D. melanogaster* and *C. elegans* cannot survive very long at 37°C [47]. This could represent a drawback for the fruit fly as a host-model because *Drosophila* experiments are usually performed at temperatures of 21°C or 25°C. Studies with *Salmonella enterica* serovar Typhimurium have however been conducted at temperatures as high as 29°C [48]; and although the fly's life expectancy was somewhat shorten by the higher temperature, it still showed a difference between the mock and the true infection.

Results presented here clearly demonstrate the validity of the *Drosophila* pricking model in the study of Bcc virulence. As well as being a powerful tool for identifying Bcc virulence factors, *D. melanogaster* can enhance our understanding of host-pathogen interactions. The sequenced genome of the fly allows microarrays experiments to be performed [49], [50]. Fluorescent proteins, such as GFP, have also been used before to monitor *P. aeruginosa* [51], [52], *Escherichia coli* [53], and *Serratia marcescens* [54] and *S.* Typhimurium [48] proliferation in the fly among others, but also to follow the expression of its immunity factors [55], [56]. Such techniques could well be applied to the study of Bcc strains.

Surette and colleagues recently reported the use of *D. melanogaster* as a host for co-infections by *P. aeruginosa* concomitant with bacteria from the CF airways microflora [52]. They discovered that some strains, non-pathogenic on their own, became infectious when in presence of *P. aeruginosa*. Knowing that essentially no environments are colonized by only one bacterial species, *Drosophila* infections opens an exciting door in the investigation of polymicrobial interactions and could most likely be used in the same fashion with the various Bcc strains [57].

## Materials and Methods

### Bacterial strains and culture conditions

Bcc strains used in this study are listed in Table 1. *Escherichia coli* SM10 λpir (*thi-1 thr leu tonA lacY supE recA*::RP4-2-Tc::Mu Km^r^ λ*pir*) served as donor for conjugation experiments [58]. Unless stated otherwise, Bcc strains were routinely grown in tryptic soy broth (TSB) (Difco) at 37°C with shaking (240 rpm) or on TSB agar plates. When required, antibiotics were added at the following final concentrations: 75 µg/ml trimethoprim and 200 µg/ml tetracycline.

**Table 1:** *Burkholderia cepacia* complex strains used in this study.

Strains^a^	Description (location)	References
*B. cepacia* (gen. I) LMG1222	Environmental isolate, onion (USA)	[61]
*B. cepacia* (gen. I) LMG18821	CF isolate (Australia)	[61]
*B. multivorans* (gen. II) LMG16660	CF isolate, (UK)	[61]
*B. cenocepacia* (gen. III) J2315	CF isolate (UK)	[61]
*B. cenocepacia* (gen. III) LMG18830	CF isolate (Australia)	[61]
*B. cenocepacia* (gen. III) LMG19240	Environmental isolate, wheat (Australia)	[62]
*B. cenocepacia* (gen. III) LMG18829	CF isolate (USA)	[61]
*B. cenocepacia* (gen. III) K56-2	CF isolate (Canada)	[61]
*B. cenocepacia* K56-2 *zmpA*	*zmpA*::*tp,* Tp^r^	[37]
*B. cenocepacia* K56-2 *zmpB*	*zmpB*::*tp,* Tp^r^	[38]
*B. cenocepacia* K56-2 *zmpA zmpB*	*zmpA*::*tp zmpB*::pKNOCK-Tet, Tp^r^ Tet^r^	this study
*B. cenocepacia* K56-2 *cepR*	*cepR*::Tn*5*-OT182, Tet^r^	[41]
*B. cenocepacia* K56-2 *cepI*	*cepI*::pKNOCK-Tet, Tet^r^	this study
*B. cenocepacia* K56-2 *bscN*	*bscN*::pKNOCK-Tet, Tet^r^	this study
*B. cenocepacia* K56-2 SALI	*hldA*::*tp,* Tp^r^	[40]
*B. cenocepacia* K56-2 RSF12	BCAL2831::pRF103, Tp^r^	[39]
*B. cenocepacia* K56-2 RSF13	*htrA*::pRF109, Tp^r^	[39]
*B. stabilis* (gen. IV) LMG18870	CF isolate (Canada)	[61]
*B. vietnamiensis* (gen.V) LMG22486	Water treatment (USA)	[63]
*B. vietnamiensis* (gen.V) LMG18835	CF isolate (USA)	[63]
*B. dolosa* (gen.VI) LMG21819	CF isolate (USA)	[64]
*B. dolasa* (gen.VI) LMG21443	Environmental isolate, root (Senegal)	[64]
*B. ambifaria* (gen. VII) HSJ1	CF isolate (Canada)	[65]
*B. ambifaria* (gen. VII) CEP0996	CF isolate (Canada)	[66]
*B. ambifaria* (gen. VII) AU0212	CF isolate (USA)	[66]
*B. pyrrocinia* (gen. IX) LMG21824	CF isolate (UK)	[64]
*B. ubonensis* (gen. X) LMG20358	Environmental isolate, soil (Thailand)	[66]

a Genomovar status is indicated in parentheses.

Growth rates were verified with a Microbiology Bioscreen C Reader (Labsystems, Finland) in 100-well microplates using 200 µl of TSB. The optical density of the cultures was measured with the wideband 420–580 nm filter.

### Construction of mutants

A 394-bp internal fragment of *cepI* and a 553-bp fragment of *bcscN* were amplified from *B. cenocepacia* K56-2 using the following primers (Table 2): cepIF with a *KpnI* site and cepIR with a *XbaI* site for the *cepI* gene, and primers bscNF and bscNR for the *bscN* gene with the same restriction sites. The PCR products were digested with *KpnI* and *XbaI* and ligated to the *XbaI* and *KpnI* sites of the suicide vector pKNOCK-Tet [59]. The constructs were then electroporated into *E. coli* SM10 cells. The plasmids were then mobilized from SM10 into *B. cenocepacia* K56-2 cells by mating. Single-crossover insertion mutants were selected on TSB agar containing tetracycline. Plasmid insertion into the target gene was confirmed by PCR. The same procedure was used for the construction of the *B. cenocepacia zmpA zmpB* double mutant: a 547-bp internal fragment of *zmpB* was amplified from *B. cenocepacia* K56-2 using primers zmpBF with a *KpnI* site and zmpBF with a *XbaI* site. It was then cloned in the *XbaI* and *KpnI* sites of pKNOCK-Tet. *B. cenocepacia* K56-2 *zmpA* was used as the recipient strain. Single-crossover insertion mutants were selected on TSB agar containing trimethoprim and tetracycline.

**Table 2:** Primers used in this study.

Primer	Primer sequence (5′-3′)^a^
cepIF	GGGGTACCCCAGTTTCGAGCGTGACCAGTT
cepIR	GCTCTAGAGCAGACGCCCATCTACCTGCT
bscNF	GGGGTACCCCGCGAATTCATCGAGCACAG
bscNR	GCTCTAGAGCAGCTCGATCTCCTGGTA
zmpBF	GGGGTACCCCGCCGTGAACGTGTACTACCA
zmpBR	GCTCTAGAGCCTTCAGGAACGCCTTGTC

^*a*^Restriction sites designed into the primers are underlined.

### *D. melanogaster* stock and maintenance

Wild-type Oregon R flies were used throughout this study. They were maintained on standard cornmeal sucrose medium and kept in a controlled environment of 25°C and 65% humidity with a 12-hours light cycle (Percival Scientific incubator). All experiments were conducted under these conditions unless stated otherwise.

### Fly feeding assays

Oregon R wild-type flies, typically seven days old, were infected by a feeding assay modified from Chugani *et al.* [29]. Plastic vials containing 5 ml of 5% sucrose and 1.5% agar were prepared. Whatman filter disks (2.3-cm diameter) were placed inside these vials on top of the agar surface. Bacterial cultures were grown in TSB to an OD_600_ of 4. Cells were then collected by centrifuging 1 ml of each culture at 7,500× *g*, washed once with 1 ml sterile PBS/5% sucrose solution and resuspended in 70 µl sterile PBS/5% sucrose. Bacterial suspensions were added to the surface of the filter paper in the plastic vials and let to dry for 15 min.

Flies starved for food and water for 7 h were anesthetised with CO_2_ and transferred to the vials per batches of 10 to 11 for a minimum of 30 flies per experiment. They were left to feed on the bacteria, and consequent death was recorded everyday. Control vials were inoculated with 70 µl sterile PBS/5% sucrose.

### Fly pricking assays

Adult female flies of 8±2 days old were infected according to a protocol modified from Baldini *et al*. [60] and Tzou *et al.* [56]. The flies were anesthetised with CO_2_ and pricked in the dorsal thorax with a 26S-gauge Hamilton needle previously dipped in the appropriate bacterial cell suspension. Bacterial cultures were grown in TSB to an OD_600_ of 2. Cells were then collected by centrifuging 1 ml of culture at 7,500× *g*, washed once with 1 ml of sterile 10 mM MgSO_4_ supplemented with 500 µg/ml ampicillin and resuspended in 1 ml of the same buffer. The addition of ampicillin to the buffer was done to prevent a possible infection with bacteria present on the surface of the fly. For every bacterial strain to be tested, a minimum of 30 flies were pricked and subsequently distributed by groups of 9 to 11 in a plastic vial containing 5 ml of 1.5% agar and 5% sucrose. Ten controls flies were pricked with a solution of 10 mM MgSO_4_ supplemented with ampicillin. The needle was washed between every replicate with 70%-grade ethanol and rinsed with the MgSO_4_ buffer. Fly survival was scored daily.

### Bacterial growth *in vivo*

Between twenty and thirty flies were pricked with bacterial cell suspension prepared as described in the fly pricking assays section. To monitor bacterial loads of the flies during the course of an infection, the number of CFU per fly on specific days following infection were recorded as follow: flies were individually put in microfuge tubes containing 70%-grade ethanol and surface-sterilized by mixing by inversion for 1 min. They were then rinsed for 1 min. in sterile water and individually grinded in 200 µl of sterile PBS with a micropestle. The suspensions obtained were then serially diluted in 0.8% NaCl and plated on TSB agar containing 25 µg/ml gentamycin and 25 µg/ml polymyxin B. For the time point regarded as zero, flies were allowed to rest for 1 h after infection before anaesthesia and homogenization as described above. Five living flies were used for each time point in order to quantify bacterial loads.

### Statistical analysis

Survival data was analyzed using Kaplan-Meier survival curves using the GraphPrism 5.0 software. Significance between survival curves was assessed using the Log-rank (Mantel-Cox) test.

### Competitive index analyses

For these experiments, bacterial cultures were grown in TSB to an OD_600_ of 1. A 1∶1 ratio mix (approximately 10^6^ CFU/ml of wild-type and mutant) was prepared in 10 mM MgSO_4_ with 500 µg/ml ampicillin. The CFUs for the two strains used in the input were counted by plating the serial dilutions of the bacterial solution using the appropriate antibiotics so to distinguish the strains. Flies were then injected with the bacterial mixture. Ninety-six hours following infection, 8 flies were sacrificed according to the method described for the measurement of *in vivo* bacterial growth. Strain discrimination was then performed by plating the bacterial suspension on TSB with the appropriate antibiotics. TSB agar contained 25 µg/ml gentamycin to determine total bacterial number; contained 25 µg/ml gentamycin and 75 µg/ml trimethoprim for mutants carrying a trimethoprim selection (*zmp*A, *zmpB*, *zmpA zmpB*, *htrA*, *hldA* mutants), or contained 25 µg/ml gentamycin and 200 µg/ml tetracycline for *cepI*, *cepR* and *bscN* mutants selection. The competitive index (CI) is defined as the CFU output ratio of the mutant strain when compared to the wild-type strain, divided by the CFU input ratio (inoculum) of the mutant over the wild-type.

## Supporting Information

Table S1 Virulence of various *B. cenocepacia* K56-2 in different infection models.
(0.01 MB PDF)
