# Supplementary material for: Drosophila melanogaster as a Model Host for the Burkholderia cepacia Complex
Source: PLoS One. 2010 Jul 12;5(7):e11467. doi: 10.1371/journal.pone.0011467 (PMC2902503; doi:10.1371/journal.pone.0011467)
Supplement: Table S1 — Virulence of various B. cenocepacia K56-2 in different infection models. (0.01 MB PDF) [file pone.0011467.s001.pdf]

Table S1: Virulence of various *B. cenocepacia* K56-2 in different infection models.

Mutation	Virulence in *D. melanogaster*[a]	Competitive indices in *D. melanogaster*	% of wild type virulence in *C. elegans* [b]	% of wild type virulence in *G. mellonella* [c]	% Alfalfa seedlings with symptoms [d]
cepI	NS	0.42	7 ± 4	100 ± 0	100 ± 0
cepR	NS	1.67	ND	ND	ND
zmpA	SD	0.51	100 ± 11	100 ± 0	100 ± 0
zmpB	NS	0.69	100 ± 17	100 ± 0	100 ± 0
zmpA zmpB	SD	0.32	ND	ND	ND
htrA (RSF13)	NS	*ND*	100 ± 13	100 ± 0	100 0
BCAL2831 (RSF12)	NS	0.48	99 ± 21	100 ± 0	96 ± 6
bscN	NS	0.23	60 ± 24	100 ± 0	87 ± 15
hldA	SD	0.005	30 ± 37	55 ± 22	100 ± 0

[a] Virulence in *D. melanogaster* was determined from survival curves obtained by the pricking method (Figure 5), statistical significance between survival curves was assessed using the Log-rank (Mantel-Cox): NS: not significant, in regards to the virulence of the wild-tyeK56-2, SD: significantly different than the wild-type ($p < 0.0005$).

[b] Percentage of dead nematodes was determined 72 h post infection, data taken from Uehlinger *et al*. (2009).
[c] Percentage of dead larvae was determined 48 h post infection, data taken from Uehlinger *et al*. (2009).
[d] Percentage of alfalfa seedlings with disease symptoms 5 days post infection, data taken from Uehlinger *et al*. (2009) and Bernier *et al*. (2003).

References for Table S1

Bernier, S. P., L. Silo-Suh, D. E. Woods, D. E. Ohman, and P. A. Sokol. 2003. Comparative analysis of plant and animal models for characterization of *Burkholderia cepacia* virulence. Infect Immun **71:**5306-13.

Uehlinger, S., S. Schwager, S. P. Bernier, K. Riedel, D. T. Nguyen, P. A. Sokol, and L. Eberl. 2009. Identification of specific and universal virulence factors in Burkholderia cenocepacia strains by using multiple infection hosts. Infect Immun **77:**4102-10.
